# Detection of a novel *Babesia* sp. in *Amblyomma javanense*, an ectoparasite of Sunda pangolins

**DOI:** 10.1186/s13071-023-06040-4

**Published:** 2023-11-22

**Authors:** Stacy Q. Y. Chong, Darren Yeo, Nur Insyirah Aidil, Jasmine L. Y. Ong, Amy H. J. Chan, Charlene Judith Fernandez, Bryan T. M. Lim, Max D. Y. Khoo, Anna M. S. Wong, Siow Foong Chang, Him Hoo Yap

**Affiliations:** 1 Animal and Veterinary Service, National Parks Board (NParks), 1 Cluny Road, Singapore Botanic Gardens, Singapore, 259569 Singapore; 2 Wildlife Management, National Parks Board (NParks), 1 Cluny Road, Singapore Botanic Gardens, Singapore, 259569 Singapore

**Keywords:** Pangolins, *Babesia*, *Amblyomma javanense*, Tick-borne disease

## Abstract

**Background:**

*Babesia* is a protozoal, tick-borne parasite that can cause life-threatening disease in humans, wildlife and domestic animals worldwide. However, in Southeast Asia, little is known about the prevalence and diversity of *Babesia* species present in wildlife and the tick vectors responsible for its transmission. Recently, a novel *Babesia* species was reported in confiscated Sunda pangolins (*Manis javanica*) in Thailand. To investigate the presence of this parasite in Singapore, we conducted a molecular survey of *Babesia* spp. in free-roaming Sunda pangolins and their main ectoparasite, the *Amblyomma javanense* tick.

**Methods:**

Ticks and tissue samples were opportunistically collected from live and dead Sunda pangolins and screened using a PCR assay targeting the *18S* rRNA gene of *Babesia* spp. DNA barcoding of the cytochrome oxidase subunit I (*COI*) mitochondrial gene was used to confirm the species of ticks that were *Babesia* positive.

**Results:**

A total of 296 ticks and 40 tissue samples were obtained from 21 Sunda pangolins throughout the 1-year study period. *Babesia* DNA was detected in five *A. javanense* ticks (minimum infection rate = 1.7%) and in nine different pangolins (52.9%) located across the country. Phylogenetic analysis revealed that the *Babesia 18S* sequences obtained from these samples grouped into a single monophyletic clade together with those derived from Sunda pangolins in Thailand and that this evolutionarily distinct species is basal to the *Babesia* sensu stricto clade, which encompasses a range of *Babesia* species that infect both domestic and wildlife vertebrate hosts.

**Conclusions:**

This is the first report documenting the detection of a *Babesia* species in *A. javanense* ticks, the main ectoparasite of Sunda pangolins. While our results showed that *A. javanense* can carry this novel *Babesia* sp., additional confirmatory studies are required to demonstrate vector competency. Further studies are also necessary to investigate the role of other transmission pathways given the low infection rate of ticks in relation to the high infection rate of Sunda pangolins. Although it appears that this novel *Babesia* sp. is of little to no pathogenicity to Sunda pangolins, its potential to cause disease in other animals or humans cannot be ruled out.

**Graphical abstract:**

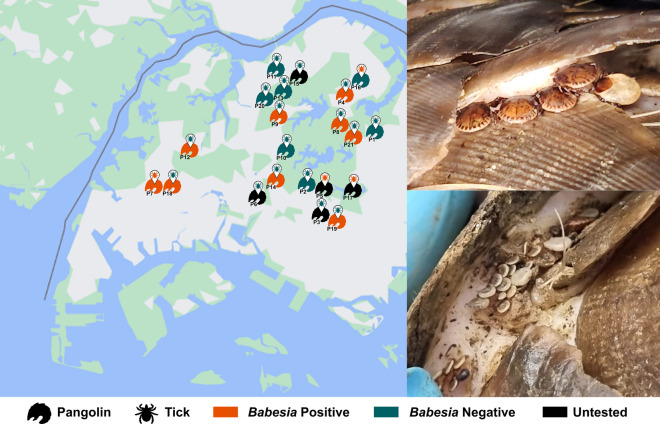

**Supplementary Information:**

The online version contains supplementary material available at 10.1186/s13071-023-06040-4.

## Background

Babesiosis is a tick-borne disease caused by intra-erythrocytic protozoan parasites of the genus *Babesia*. It is the second most prevalent haemoparasite in the world, with more than 100 species identified in a wide range of vertebrate hosts [1]. Various wildlife species have been identified as reservoir hosts of *Babesia* and are key in the maintenance and transmission of the parasite [2–4]. As deforestation and climate change cause shifts in host ranges and increased human-wildlife contact [5], the discovery and monitoring of potentially zoonotic pathogens in wildlife hosts, as well as identifying their vectors, are becoming paramount.

In general, wildlife reservoirs for babesiosis and their vectors are poorly studied compared to domestic hosts owing to difficulties that make biosurveillance challenging [6]. This dearth of data is even more drastic in the tropics, with monitoring efforts mostly focused on economically significant domestic cattle [7]. This is particularly concerning given that hotspots for mammalian species richness are mostly found there [8]. It is also difficult to make management decisions using data from temperate and subtropical regions, which have very different climates and environmental characteristics. Furthermore, insufficient data are available on the distribution, host specificity and reservoir competency of tropical tick vectors. While it has been largely established that canine babesiosis is endemic in Asia and predominantly caused by *Babesia gibsoni* and *B. canis*, relatively little is known about the diversity or prevalence of *Babesia* in wildlife in Southeast Asia.

The Sunda pangolin is native to Singapore and the rest of Southeast Asia but is poorly understood given the historical lack of research interest as well as its elusive and nocturnal behaviour [9]. Over the past few decades, they have been threatened by over-exploitation for their scales and meat, which find their way into traditional medicines in East Asian markets [10], and consequently have been classified as critically endangered by the IUCN Red List of Threatened Species [11]. Like other wildlife species indigenous to Southeast Asia, there is a paucity of information regarding the blood parasites infecting Sunda pangolins. Researchers in Thailand, however, recently reported a novel *Babesia* species detected in samples obtained from confiscated pangolins [12]. In our study, we screened both the Sunda pangolin and the ticks (*Amblyomma javanense*) found on them for this novel *Babesia* sp. and highlight *A. javanense* as a potential vector for pangolin babesiosis.

## Methods

### Sampling of Sunda pangolins

A total of 25 free-ranging Sunda pangolins in Singapore were retrieved in 2022. Twenty of these pangolins were dead upon sampling, with traumatic injuries found in 18 of them. Tissue samples such as kidney, liver and spleen were opportunistically collected depending on the integrity of the carcass. In the absence of viable organ tissue, a muscle sample was collected instead. The remaining pangolins were live individuals that were part of rescue and release operations. Where possible, an EDTA blood sample was obtained from the live animal. All tissue samples were stored at – 20 °C pending DNA extraction.

For both live and dead pangolins, the skin beneath the scales was examined and ticks were removed and placed in 70% ethanol. Each tick was morphologically observed using a stereomicroscope (Olympus SZ51, Japan) and its species, life stage, sex and feeding status were recorded [13].

### DNA extraction of tick and tissue samples

All adult or engorged ticks were processed individually, while unfed nymphs were pooled in groups of up to five according to their host. Larvae were not processed. Each tick was surface sterilised in 70% ethanol to remove any surface dirt, and any residual host tissue on the tick’s mouthparts was detached using blunt forceps and discarded. The tick specimens were then air-dried before transfer to a 1.5-ml microcentrifuge tube and dissection into smaller pieces using a sterile scalpel blade. DNA extraction was performed using the DNeasy^®^ Blood and Tissue kit (QIAGEN, Germany) according to the manufacturer’s instructions.

For pangolin tissue samples, organs were sectioned into pieces of approximately 10 to 15 mg, while EDTA blood (200 µl) was aliquoted for extraction. Following this, the samples were subjected to the same extraction protocol used for the ticks. The eluted DNA was stored at − 20 °C pending further testing.

### PCR and Sequencing

All DNA extracts were screened for *Babesia* spp. using a primer pair BJ1/BN2 (see Additional file 1: Table S1), targeting an approximately 400–500 base pair (bp) partial region of the piroplasmid *18S* ribosomal (r) RNA. The PCR reaction was set up in a 25-µl reaction mixture containing 5 µl DNA template, 1X Promega Green GoTaq^®^ Flexi Buffer, 0.1 mM of each dNTP, 2 mM of MgCl_2_, 350 nM of forward and reverse primers and 1 U of GoTaq DNA polymerase (Promega, USA). Thermal cycling conditions were set as follows on a ProFlex PCR System (Thermo Scientific, USA): initial denaturation at 95 °C for 2 min, followed by 35 cycles of activation at 95 °C for 30 s, annealing at 58 °C for 45 s and elongation at 72 °C for 45 s, ending with a final extension at 72 °C for 5 min. PCR products were visualised using a 1.5% agarose gel. Amplicons were sent to a commercial company (Bio Basic Asia Pacific Pte Ltd, Singapore) for PCR clean-up and Sanger sequencing to obtain bidirectional sequences. In cases where sequencing failed to obtain a clean chromatogram, PCR was repeated with an annealing temperature of 60 °C using the genus-specific *18S* rRNA primer pair BabsppF/BabsppR.

If *Babesia* sp. was detected, the sample was subjected to a second PCR using the primer pair Piro18S_Frag1F/ Piro18S_Frag2R to amplify the full length *18S* rRNA sequence. PCR cycling parameters were set as described by Baneth et al. [19]. For tick samples that were PCR-positive, DNA barcoding using the cytochrome oxidase subunit I mitochondrial (*COI*) gene was also performed to confirm the tick species using the protocol outlined in Hajibabaei et al. [14].

### Data analyses

The sequences were processed and trimmed using Geneious Prime [15] to remove priming sites and unreliable regions. The edited sequences were compared to sequences deposited in GenBank using the Basic Local Alignment Search Tool (BLAST) to obtain species identifications (> 97% identity match).

Using GenBank’s Taxonomy search (“Babesia”) and the “18S” search term, a list of *Babesia 18S* sequences were obtained. A cut-off threshold of > 1000 bp was used to obtain sufficient informative sites for a phylogenetic analysis. The longest sequence of each named species was used as a species representative for downstream analyses. These species names (see Additional file 2: Table S2) were then used in GenBank’s Taxonomy search and all matches from each species were downloaded in the “GenBank (full)” format, which retains all metadata associated with the sequence. A custom script was written to identify and report all cases where the “host” field had an entry. This was used to classify whether each *Babesia* species had a known host from the following vertebrate taxa: Carnivora, Ungulata, Primates, Rodentia, Aves and Other Mammals. The hosts were also classified as domestic or wild where applicable. Cases of human infections were classified under “domestic primates”.

The *18S* sequences generated in this study were aligned with the afore-mentioned representative sequences from GenBank (Additional file 2: Table S2) as well as an outgroup *Cardiosporidium cionae* (EU052685). The alignment was performed in MAFFT v7 [16] under default parameters except with the “adjust direction according to the first sequence” selected. A maximum likelihood (ML) tree was generated from the aligned sequences in raxmlGUI 2.0 [17] with the GTR GAMMA model and rapid bootstrapping with 1000 bootstrap replicates.

## Results

### Detection of *Babesia* sp. in ticks and pangolin tissue

Throughout the 1-year study period (2022), a total of 296 ticks and 40 tissue samples were obtained from 21 Sunda pangolins. Four pangolins were excluded from this study because of extensive decomposition of the carcass and the absence of intact ticks upon examination. The number of ticks collected from each pangolin ranged from 2 to 60 for the carcasses and 3 to 25 for the live animals. The ticks were most commonly located under the scales near the head and upper torso of the pangolin. Of the 296 ticks collected, 149 were adults and 147 were nymphs. All ticks were morphologically identified as *A. javanense* and confirmed via *COI* DNA barcoding (sequences deposited in GenBank: OR229763-OR229767). DNA markers of novel *Babesia* sp. were detected in five ticks collected from four different pangolins (Table 1), all of which were extracts of individual specimens. Of the five tick detections, two were in engorged nymphs from an infected pangolin and likely detected through the tick’s blood meal. The remaining three male ticks appeared unfed, and the mammalian *cytochrome b* (*cyt b*) gene was not detected in these samples when screened with CYTB GVL14724/CYTB H15149 primers [18].

**Table 1:** Detection of *Babesia* sp. in Sunda pangolins and *Amblyomma javanense* ticks

Pangolin no.	Status at point of sampling	Sex	Ticks		Pangolin tissue	
			Total sampled (pools tested)	*Babesia* sp. detections	Total	*Babesia* sp. detections
1	Dead	F	5 (5)	0	4	0
2	Dead	U	3 (3)	0	1	0
3	Live	M	16 (15)	0	–	–
4	Dead	M	5 (5)	0	1	1
5	Live	M	11 (11)	1	–	–
6	Live	M	6 (6)	0	–	–
7	Dead	M	44 (29)	2	4	4
8	Dead	M	15 (13)	0	3	3
9	Dead	F	8 (8)	0	5	1
10	Dead	M	7 (6)	0	2	0
11	Dead	M	5 (5)	0	1	0
12	Live	M	25 (19)	0	1	1
13	Dead	U	2 (2)	0	1	0
14	Dead	F	17 (17)	0	2	2
15	Live	M	3 (3)	0	–	–
16	Dead	M	23 (21)	1	1	0
17	Dead	F	14 (8)	1	–	–
18	Dead	M	22 (21)	0	3	2
19	Dead	M	2 (2)	0	5	4
20	Dead	M	3 (3)	0	3	0
21	Dead	M	60 (29)	0	3	1
Total			296 (231)	5	40	19

*M *male, *F *female, *U *undetermined

Of the 40 tissue samples collected, 39 were organ samples obtained from pangolin carcasses and one was a blood sample obtained from a rescued live pangolin. *Babesia* sp. was detected in 19 extracts, including those from the blood and organs such as the spleen, liver, kidney, lung, intestines, heart and skeletal muscle (Table 1). The protozoa were detected in at least one tissue sample from nine Sunda pangolins, indicating that approximately half the pangolins with tissues sampled (52.9%: 9 of 17) were infected with the same species of *Babesia*.

**Fig. 1:**
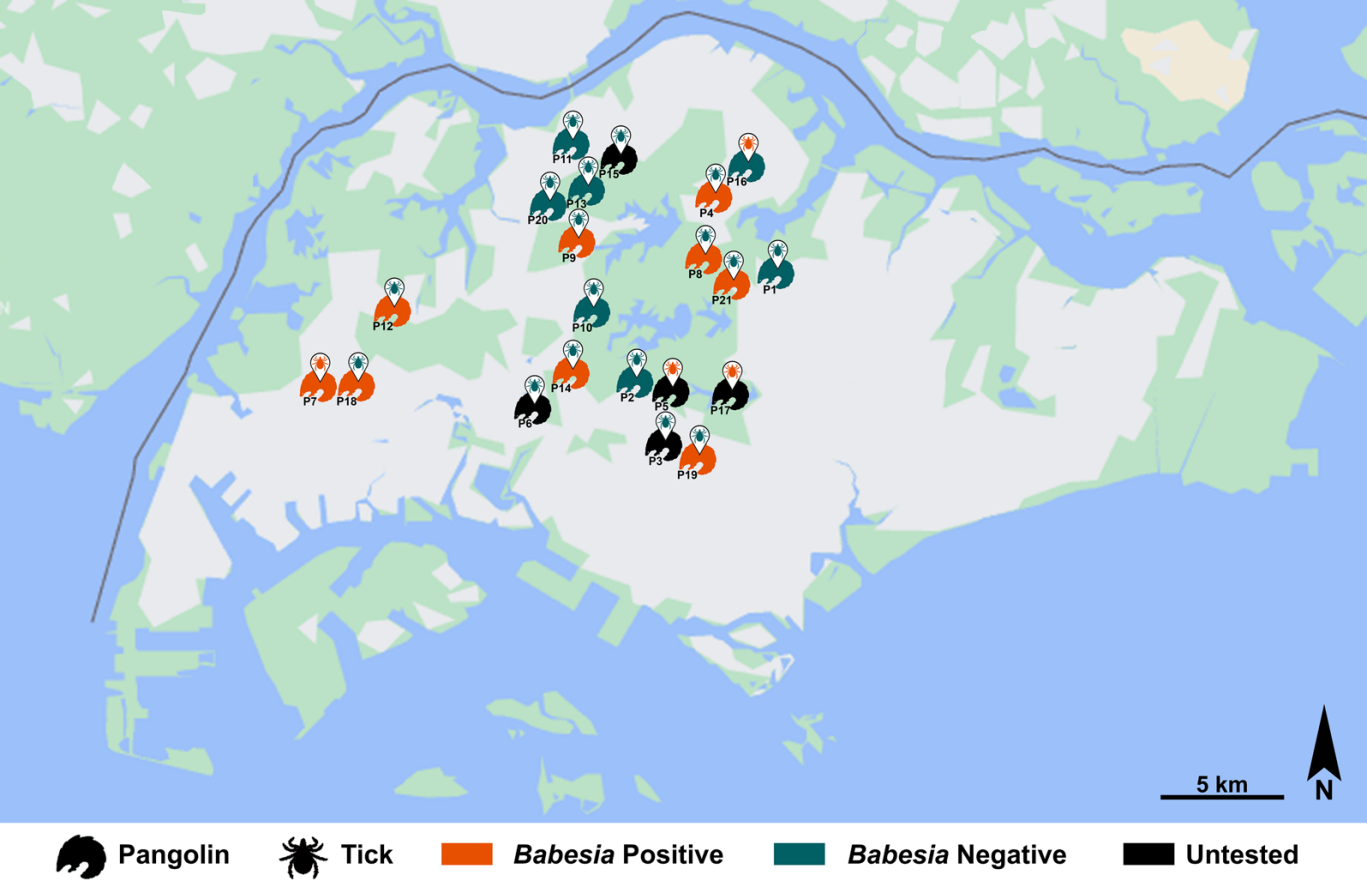
Spatial distribution of *Babesia* detections in either pangolin or tick in Singapore. The pangolin numbers (P1 to P21) indicated in the map refer to the pangolin numbers in Table 1

Based on the geographic location where the pangolin carcass or live animal was found, it appears that the parasite, harboured by either the pangolin or the tick, is widely distributed across the pangolin’s range in Singapore, with detections in the west, north, central and northeast regions of the island (Fig. 1) but with no distinct foci. Sunda pangolins are rarely found in the southern and eastern parts of mainland Singapore. As such, most of the carcasses in this study were retrieved around the central water catchment forest and none from the east.

Partial *18S* rRNA sequences obtained from all samples using BJ1/BN2 or BabsppF/BabsppR shared a 99.76–100% similarity to unclassified *Babesia* sp. sequences deposited in GenBank under accession numbers KX168695, KX168696, MZ173490, MZ173491, MZ173492, MZ173493, MZ173494, MZ173495 and MZ173496. These nine *Babesia* sp. sequences were all isolated from Sunda pangolin blood samples, with the first two from samples collected in 2013 by the Veterinary Research Institute in Malaysia (unpublished) and the remaining from samples collected in 2017 by Wildlife Quarantine Centres in Thailand [12].

### Molecular phylogenetics

Close to full-length (> 1.4 kbp) *18S* rRNA sequences were obtained from three samples and partial (550–1.4 kbp) *18S* fragments were obtained from another three samples using the primers Bab18S_Frag1F and Bab18S_Frag2R [19]. BJ1 and BN2 primers [20] yielded partial *18S* rRNA sequences (450–500 bp) from another eight samples, while BabsppF and BabsppR primers [21] yielded partial *18S* sequences (380–520 bp) from another nine samples. Sequences were deposited in GenBank (OR229733 to OR229755).

**Fig. 2.**
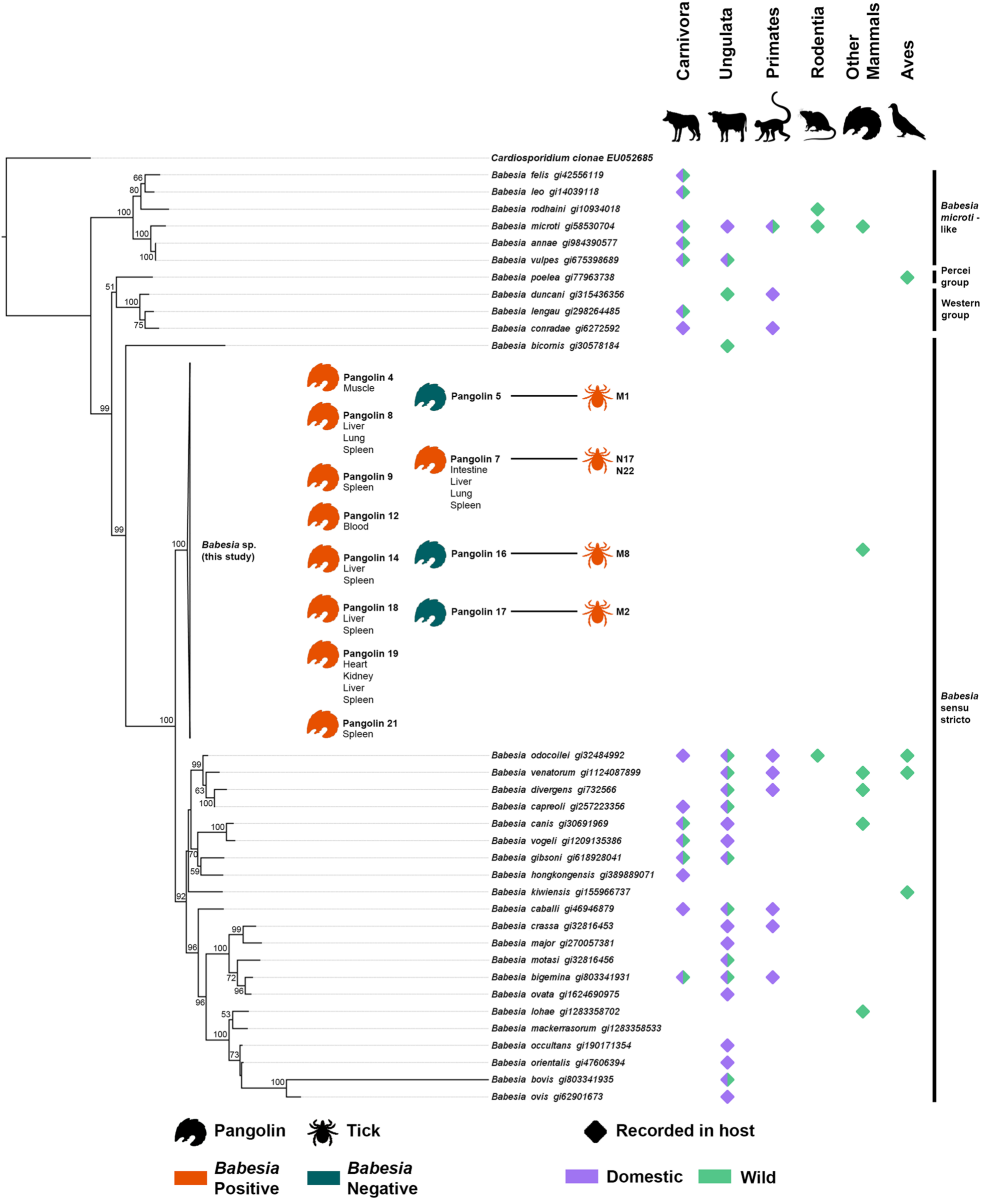
*18S* ML tree of *Babesia* species indicates the evolutionarily unique position of the Sunda pangolin *Babesia* sp. Clade names are adapted from Jalovecka et al. 2019 [22]

After alignment to the other *Babesia* sequences from GenBank, a 1830-bp matrix was generated and used for phylogenetic analyses. The ML tree (Fig. 2) has 20/53 nodes with 80 or more bootstrap support (37.7%) and 35/53 with 50 or more (66.0%). In this tree, all novel *Babesia* sequences from our study, Malaysia and Thailand grouped into a single monophyletic clade. As such, they were collapsed in the final tree under “*Babesia* sp. (this study)". It is placed at the base of the *Babesia* sensu stricto clade along with *B. bicornis* with high support (100 bootstrap support). Placement of the four clades corresponds to the literature [22]. The tree also does not appear to have multi-species clades that have detections only in wildlife hosts.

## Discussion

This is the first report documenting the detection of a *Babesia* species in *A. javanense* ticks. In China, Zhai et al. [23] screened 224 *A. javanense* ticks collected from 21 confiscated Sunda pangolins for a range of tick-borne pathogens including *Babesia*, but none were detected except for *Ehrlichia*. Other studies on *A. javanense* ticks focused on bacterial and viral testing and identified novel *Anaplasma* sp., *Borrelia* sp. and *Pestivirus*, suggesting that this tick species could be a competent vector for a variety of pathogens that have yet to be fully characterised [24–26].

This study is also the first demonstration of this novel *Babesia* sp. in the pangolin tick as well as its host, *M. javanica*. Yodsheewan et al. [12] first detected this *Babesia* species in the blood of confiscated Sunda pangolins housed at different rehabilitation centres across Thailand and implied that the parasite was widely distributed across the country, with 41.4% of tested pangolins infected. A similar phenomenon was observed in this study, wherein the novel *Babesia* sp. was detected in 52.9% of pangolins sampled across various locations in Singapore. This *Babesia* species was reported to be detected in samples collected as early as 2013 in Malaysia (Koh et al., unpublished); therefore, it is probable that this tick-borne parasite is now well established in Southeast Asia’s Sunda pangolin populations.

Although approximately half of the screened pangolins were infected, the novel *Babesia* sp. was only detected in a small portion of ticks infesting the pangolins at a minimum infection rate of 1.7%. This is comparable to global infection rates estimated from two separate meta-analyses by Karshima et al. [27] and Onyiche et al. [28] on the prevalence of *Babesia* sp. in tick vectors. While the former focused on zoonotic *Babesia* sp. and the latter on questing ticks, both derived similar values of 2.1% and 2.2% respectively on the infection rate of ticks worldwide. It should also be considered that most tick specimens in this study were obtained from carcasses, and ticks have been shown to spontaneously detach themselves from the host after death because of a drop in body temperature and the cessation of peripheral blood flow [29]. Furthermore, all ticks collected from the carcasses were dead upon sampling and it is possible that nucleases released from the ticks upon death might have caused some degree of DNA degradation, thereby potentially accounting for the marginally lower detection rate.

The seemingly high incidence of novel *Babesia* sp. in *M. javanica* despite a low infection rate of ticks could be a result of the host specificity of the tick vector. Specialist ticks which have a restricted specificity to or a high preference for certain host species are more likely to efficiently transmit the parasite within the host population, in contrast to generalist ticks which would instead be more likely to introduce the parasite into new host species [30]. Although the ecology of *A. javanense* is not fully elucidated, it has been suggested to be a highly host-specific and nidicolous species, adapted to the habitat used by pangolins [13]. In addition to this study, other parasitological surveys on *M. javanica* have only reported a single tick species, *A. javanense*, found on the pangolins sampled [23–26, 31]. This would support the hypothesis that *A. javanense* is the vector responsible for transmission of this *Babesia* species, resulting in the apparent high prevalence observed in *M. javanica* in Singapore and Thailand. However, to confirm this hypothesis, vector competence trials with controlled variables will be required to effectively demonstrate the transmission of this *Babesia* species from tick to host and vice versa.

Besides the feeding habits of the tick vector, another possible explanation for the high pangolin infection rate would be vertical transmission of *Babesia* sp. from an infected female pangolin to its offspring. Although the exact mechanism by which *Babesia* in maternal red blood cells is able to breach the placental barrier is still unknown, recent studies have provided evidence of in utero transmission of multiple *Babesia* species including *Babesia microti, B. caballi*, *B. canis* and *B. gibsoni* in various mammalian species [32–36]. For example, in the white-footed mouse, Tufts et al. [37] demonstrated a transmission efficiency of 74% for *B. microti* from mother to foetus, suggesting that transplacental transmission plays an important role in maintaining the parasite in the reservoir population.

As more than three-quarters of the pangolins included in this study were carcasses with varying degrees of trauma and decomposition, post-mortem examinations had limited value in providing data on the pathogenicity of this *Babesia* sp. Yodsheewan et al. [12], however, were able to conclude that there was no correlation between the *Babesia* infection and the pangolin’s health based on haematological analyses of live infected and non-infected pangolins. Additionally, the examinations of peripheral blood films revealed low parasite levels in infected pangolins. This is unsurprising as most *Babesia* sp. cause subclinical infections in their natural wildlife host, enabling the persistence of the parasite in the host population. The chronic, low parasitaemia allows host survival and also extends the time period in which a tick may acquire the parasite from the host [30]. Nonetheless, it should be noted that the introduction of *Babesia* into a naïve host species could potentially result in clinical disease in the maladapted host which is unable to muster an adequate immune response. This might occur through a tick vector that can parasitise exceptional hosts, as in the case of *A. javanense*. In rare circumstances, it has been discovered on non-pangolin species such as a wild boar, an Asian water monitor and a mouse-deer, all of which also inhabit Singapore [38–40].

This novel *Babesia* sp. appears to be evolutionarily distinct, having few close relatives in our *18S* ML tree (Fig. 2), as well as in the tree published by Yodsheewan et al. [12]. This is expected given that their host species is also deemed evolutionarily distinct [41] and is supported by the “EDGE of Existence” conservation programme that prioritises evolutionary and genetic diversity of endangered species [42]. It is thus likely that this novel *Babesia* co-evolved with its pangolin host, lending further credence to *M. javanica* being its natural host. *Amblyomma javanense* also appears to share this property in the literature, although there does not seem to be a consensus on its placement [23, 43, 44]. This possibly indicates a tightly knit co-evolutionary and co-dependent system of parasite, vector and host, which can be threatened by host co-extinction [45].

Evolutionarily distinct parasites that have co-evolved with their hosts are not rare. Other such parasites were also featured on our *18S* ML tree (Fig. 2), such as *Babesia poelea* found on brown boobies [46], *B. bicornis* on black rhinoceros [47] and *B. kiwiensis* on brown kiwis [48]. There are probably many other undiscovered *Babesia* species in wildlife hosts, but such data are unfortunately scant, as *Babesia* screening is primarily focused on domestic animals. In contrast to the tree from Yodsheewan et al. [12], our *Babesia* tree reconstructed from full *18S* sequences (Fig. 2) does not reveal any clade that is exclusive to wildlife species, which suggests that host shifts are not uncommon in *Babesia* and are a frequent occurrence in parasite evolution [49].

## Conclusion

Further investigation is required to confirm that *A. javanense* is responsible for the transmission of this novel *Babesia* species in Sunda pangolins. Nonetheless, increased biosurveillance of wildlife and their associated ticks in the tropics is crucial given the zoonotic potential of *Babesia* spp. and the increasing human-wildlife contact brought about by deforestation and climate change. It is therefore imperative that greater emphasis be placed on screening wildlife for novel parasites, with mammalian Evolutionarily Distinct and Globally Endangered (EDGE) species [41] being a good place to start.

### Supplementary Information

**Additional file 1: Table S1. **Primer pairs used in this study.

**Additional file 2: Table S2. ***Babesia *species used in phylogenetic analyses.

## Data Availability

All data generated or analysed during this study are included in this article and supplementary information files.
