# Supplementary material for: Detection of a novel Babesia sp. in Amblyomma javanense, an ectoparasite of Sunda pangolins
Source: Parasit Vectors. 2023 Nov 22;16:432. doi: 10.1186/s13071-023-06040-4 (PMC10664631; doi:10.1186/s13071-023-06040-4)
Supplement: Supplementary file 1 — Additional file 1: Table S1. Primer pairs used in this study. [file 13071_2023_6040_MOESM1_ESM.docx]

**Additional File 1: Table S1**. Primer pairs used in this study

| Primer name | Primer sequence (5’ - 3’) | Target gene | Amplicon size | Reference |
| --- | --- | --- | --- | --- |
| BJ1 | GTCTTGTAATTGGAATGATGG | *18S* rRNA of piroplasmids | 400 to 500 bp | [1] |
| BN2 | TAGTTTATGGTTAGGACTACG |  |  |  |
| BabsppF | GTTTCTGMCCCATCAGCTTGAC | *18S* rRNA of *Babesia spp.* | 422 to 440 bp | [2] |
| BabsppR | CAAGACAAAAGTCTGCTTGAAAC |  |  |  |
| Piro18S_Frag1F | GTTGATCCTGCCAGTAGT | *18S* rRNA of piroplasmids | 1500 to 1700bp | [3] |
| Piro18S_Frag2R | AACCTTGTTACGACTTCTC |  |  |  |
| LepF | ATTCAACCAATCATAAAGATATTGG | *COI* of invertebrates | 658bp | [4] |
| LepR | TAAACTTCTGGATGTCCAAAAAATCA |  |  |  |

**References**

1. Casati S, Sager H, Gern L, Piffaretti J-C. Presence of potentially pathogenic *Babesia* sp. for human in *Ixodes ricinus* in Switzerland. Annals of agricultural and environmental medicine. 2006;13:65–70.

2. Hilpertshauser H, Deplazes P, Schnyder M, Gern L, Mathis A. *Babesia* spp. Identified by PCR in Ticks Collected from Domestic and Wild Ruminants in Southern Switzerland. Appl Environ Microbiol. 2006;72:6503–7.

3. Baneth G, Nachum-Biala Y, Birkenheuer AJ, Schreeg ME, Prince H, Florin-Christensen M, et al. A new piroplasmid species infecting dogs: morphological and molecular characterization and pathogeny of *Babesia negevi* n. sp. Parasites & vectors. 2020;13:1–13.

4. Hajibabaei M, Janzen DH, Burns JM, Hallwachs W, Hebert PDN. DNA barcodes distinguish species of tropical Lepidoptera. Proc Natl Acad Sci USA. 2006;103:968–71.
